# Demographic factors associated with healthcare avoidance and delay in the transgender population: Findings from a systematic review

**DOI:** 10.1016/j.dialog.2023.100159

**Published:** 2023-11-14

**Authors:** Siobhan D. Thomas, Robert King, Mike Murphy, Maria Dempsey

**Affiliations:** School of Applied Psychology, University College Cork, Cork Enterprise Centre, North Mall, Cork, Ireland

**Keywords:** Transgender, LGBTQIA, Healthcare avoidance and delay, Healthcare utilization, Patient engagement

## Abstract

**Purpose:**

Healthcare avoidance and delay (HAD) in the transgender population has been well documented, and research has explored a range of associated factors that help to identify those most at risk of HAD. This review addresses a gap in the research by synthesizing research exploring associations between HAD and demographic factors.

**Methods:**

A systematic search of literature published at any time up to December 2021 was conducted, using five databases (EBSCO, EMBASE, PubMed, Scopus, and Web of Science) and manually searching reference lists of included studies. After exclusion of duplicates, 608 unique records were subjected to double screening. Papers reporting statistical analyses of HAD in association with any sociodemographic variables were included in this review. Papers consisted of nineteen cross-sectional studies. Narrative synthesis was used to address findings.

**Results:**

Nineteen studies met inclusion criteria, exploring HAD in association with a wide range of demographic factors, including sex and gender, social transition factors, age, race and ethnicity, socioeconomic factors, veteran status, education, sexuality, relationship status, citizenship, place of residence, and state demographics. Findings identified intra-community demographic risk factors, with consistent evidence for increased HAD among transmasculine, and younger, participants. Lower income and higher educational attainment were also associated with increased HAD, while remaining areas had weak or little evidence for association with HAD.

**Conclusion:**

This review expands knowledge in this area by highlighting demographic factors associated with increased HAD in research literature, and exploring how these may be further investigated to address substantial gaps in the body of research.

Transgender (trans) people report reduced utilization of health services [1,2]. While healthcare utilization for transgender people may be negatively impacted by scarcity of accessible health services, so too may it relate to a direct avoidance or postponement of healthcare use. Healthcare Avoidance and Delay (HAD) can be defined as the avoidance, non-use, postponement, or delay of health care services, and is well established as a prominent barrier in transgender health research [1,3]. HAD is often suggested to be a contributing factor in transgender health disparities [4], which are found in areas of both physical and mental health [5,6].

Studies examining factors associated with HAD offer valuable insight into possible explanatory and concurrent mechanisms, helping researchers and policymakers identify those most at risk of this behavior. Commonly identified are external sources of minority stress, as previous negative experiences in healthcare and a lack of knowledgeable healthcare providers are commonly reported as barriers to healthcare use [1,7,8]. In a previous review, we found evidence for association between HAD and a range of minority stress factors, both in individual experiences of healthcare and in broader systemic barriers [9].

However, a notable gap exists in understanding how individual level factors may also play a role in HAD, or indeed how they may act as mediating or moderating variables in the relationship between HAD and other barriers. Of particular interest are sociodemographic factors, which are important in understanding occurrences of HAD within the transgender population. While transgender status itself is a risk factor for reduced help-seeking in some domains [10,11], research has also found variance in transgender HAD across demographic factors such as gender identity [12], age [13] and income [3], indicating that such intra-community demographic factors are likely to constitute further risk or protective factors. Additionally, transgender people share many of the same social determinants of health as cisgender counterparts, such as race, ethnicity and socioeconomic status. As such, many transgender people experience intersecting forces of marginalization, which may result in intra-community differences based on such demographic characteristics. Identifying intersecting power relations greatly improves our understanding of health disparities in trans health research [14], and a synthesis of research in this area will provide insights into the body of evidence for demographic risk factors, identifying subsections of the transgender population most at risk, exploring potentially intersecting forms of marginalization, and identifying where interventions may be most needed.

A synthesis of the body of evidence in this area has not yet been published. This review will synthesize current knowledge of intra-community demographic differences in HAD, and thus will advance the state of the art by establishing gaps and limitations in the research body and offering recommendations for future research. As HAD measurement varies substantively in research literature, this review also explores the effect of variation in measurement of HAD on the body of evidence, contributing to a comprehensive understanding of demographic variation across the concept of HAD in general.

## 1 Methods

This review is part of a broader process of systematically reviewing research measuring any variables in association with HAD in transgender populations, the protocol for which was registered on PROSPERO [15]. Inclusion and exclusion criteria for this review are outlined in Table 1. Ethics committee approval was not required for this review and this paper is reported in accordance with Preferred Reporting Items for Systematic Reviews and Meta-Analysis guidelines [16].

**Table 1:** Inclusion and Exclusion Criteria.

	Inclusion Criteria	Exclusion Criteria
Population	Transgender participants of any age and from any country, defined by self-identification of discordance between gender identity and birth sex	No transgender participants No separate analysis for transgender participants
Exposure:	Any demographic variable	Non-demographic variables
Outcome:	Healthcare Avoidance or Delay defined as: explicit avoidance/delay/non-use/disengagement/postponement in any healthcare settings	Medication adherence Treatment adherence
Study Design	Quantitative and mixed methods studies, of any design, using statistical analysis to measure group differences of, or associations with, the outcome variable	Qualitative Studies Quantitative or mixed methods studies that report only descriptive statistics, with no testing between variables

### 1.1 Information sources

Five databases were searched for records: EBSCO, EMBASE, PubMed, Scopus, and Web of Science, and reference lists of included studies were searched. No restrictions on dates or locations were introduced.

### 1.2 Search strategy

Standardized keywords, supplemented by controlled vocabulary of indexed subject headings for several databases, were used to identify relevant literature. Table 2 outlines full search terms for each database.

**Table 2:** Database Search Terms.

EBSCO	(MM “Transgender Persons”) OR transgender OR “gender nonconforming” OR non-binary OR transsexual) AND (avoid* OR delay* OR nonus* OR non-us* OR disengag* OR postpon*) AND (healthcare OR health-care OR “health care” OR “medical care”) AND (predic* OR determin* OR correlat* OR caus* OR antecedent* OR relation* OR regress* OR associat* OR mediat*) Note: “Scholarly (Peer Reviewed) Journals” selected to narrow results
EMBASE	(‘transgender’/exp. OR ‘sexual and gender minority’/exp. OR ‘transsexual’/de OR ‘gender nonconforming’ OR ‘non-binary’) AND (avoid* OR delay* OR nonus* OR non-us* OR disengag* OR postpon*) AND (healthcare OR health-care OR “health care” OR “medical care”) AND (predic* OR determin* OR correlat* OR caus* OR antecedent* OR relation* OR regress* OR associat* OR mediat*)
PubMed	(“Sexual and Gender Minorities”[Mesh]) OR transgender OR non-binary OR “gender nonconforming” OR transsexual AND (avoid* OR delay* OR nonus* OR non-us* OR disengag* OR postpon*) AND (healthcare OR health-care OR “health care” OR “medical care”) AND (predic* OR determin* OR correlat* OR caus* OR antecedent* OR relation* OR regress* OR associat* OR mediat*)
Scopus	(KEY(transgender OR “non-binary” OR “gender nonconforming” OR transsexual)) AND (KEY(avoid* OR delay* OR nonus* OR non-us* OR disengag* OR postpon*)) AND (KEY(healthcare OR “health-care” OR “health care” OR “medical care”)) AND (predic* OR determin* OR correlat* OR caus* OR antecedent* OR relation* OR regress* OR associat* OR mediat*) Note: KEY operator used to indicate keywords and to narrow search results
Web of Science	TS = (transgender OR transsexual OR “gender nonconforming” OR non-binary) AND TS = (avoid* OR delay* OR nonus* OR non-us* OR disengag* OR postpon*) AND TS = (healthcare OR health-care OR “health care” OR “medical care”) AND TS = (predic* OR determin* OR correlat* OR caus* OR antecedent* OR relation* OR regress* OR associat* OR mediat*) Note: TS operator used to indicate keywords and to narrow search results

### 1.3 Selection process

All records were independently double-screened at both title and abstract, and full-text phases. Disagreements between reviewers were resolved through discussion.

### 1.4 Data collection and data items

Data extracted consisted of statistical information for variables measuring HAD and for all statistical tests in relation to HAD. Corresponding authors were contacted where further detail or clarification was required. Where sufficient statistical information was provided, effect sizes and confidence intervals were calculated when not reported in studies.

### 1.5 Quality assessment

To determine methodological quality and risk of bias, the JBI Checklist for Analytical Cross-Sectional Studies was used [17]. Item 4, “Were objective, standard criteria used for measurement of the condition?” was removed, as self-identified transgender status was the basis for participant recruitment in studies. Quality assessment was conducted in duplicate by three reviewers, and was used to inform synthesis by highlighting methodological concerns in the body of research.

### 1.6 Synthesis methods

Findings are addressed through narrative synthesis, as heterogeneity of included studies, overlap of data sources, and limited statistical information provided by authors made meta-analysis and subgroup analysis unfeasible. For studies reporting similar bivariate and multivariate results, or studies reporting similar results across multiple regression models, only statistical data related to final multivariate results and final model results are reported. Where studies report differences in results between bivariate and multivariate analyses, or notable variance between regression models, relevant statistical data is reported and implications are addressed.

## 2 Results

**Fig. 1:**
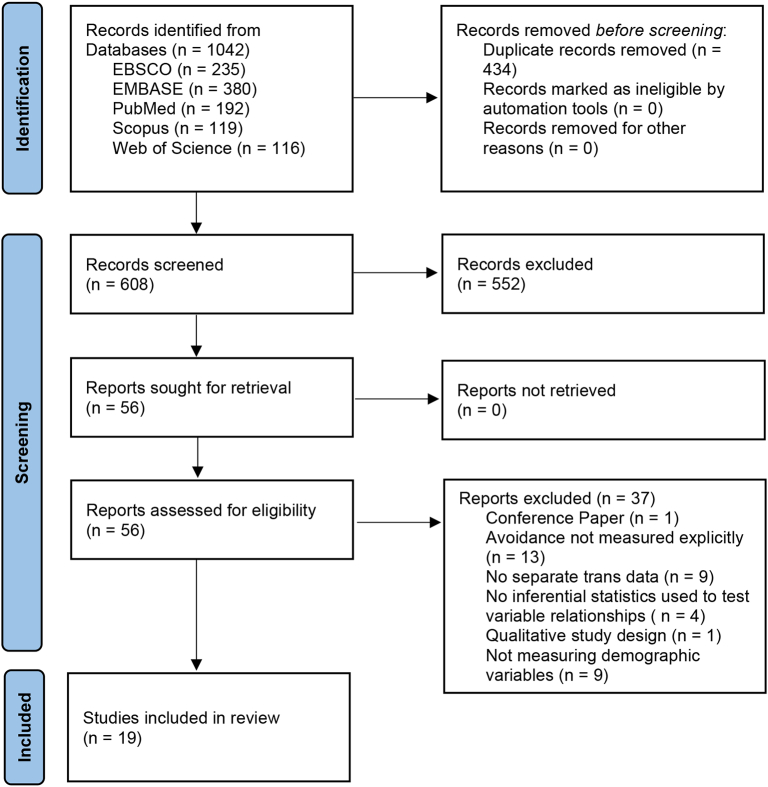
*PRISMA* flow diagram detailing screening and selection process.

Fig. 1 outlines the screening and study selection process. Of 608 unique records, 28 studies met inclusion criteria in the larger review process measuring any variables in relation to HAD, and 19 of these met inclusion criteria for this review due to inclusion of demographic variables. Details of included studies are outlined in Table 3.

**Table 3:** Characteristics of Included Studies.

Study	Country	Data Source	Sample	Participants (N)	Exposure Variables	Analytic Design
Aboussouan et al. (2019)	United States	Trans Lifeline Mental Health Survey	Transgender adults (18+)	4285	Veteran status, Age, Natal Sex Outside Gender Binary, Racial Minority	Bivariate associations
Bauer et al. (2014)	Canada	Trans PULSE Project	Transgender people (16+)	167	Gender spectrum	Bivariate association (using estimates calculated by respondent-driven sampling tool)
Cruz (2014)	United States	National Transgender Discrimination Survey	Transgender adults (18+)	4049	Gender spectrum, Transsexual identity, Genderqueer Identity, Race/ethnicity, Age, Relationship status, Income	Binary logistic regression and multinomial logistic regression
Feldman et al. (2021)	United States	TransPop and U.S. Trans Survey	Transgender adults (18+)	TransPop: 271 USTS: 26864	Gender spectrum, Age	Bivariate analyses and logistic regression
Glick et al. (2018)	United States	National Transgender Discrimination Survey	Transfeminine adults (18+)	2248	Education, Income, Age, Race/ethnicity, Relationship Status	Bivariate analyses and multivariate logistic regression
Goldenberg et al. (2019)	United States	Affirming Voices for Action Project	Black transgender youth (16–24)	110	Age, High school education, Gender spectrum	Bivariate analyses and multivariate logistic regression
Goldenberg et al. (2020)	United States	U.S. Transgender Survey	Transgender adults (18+)	23,323	State proportion of non-Hispanic white people, State population density, State proportion living in a rural area, State proportion living in an urban area, Age, Gender spectrum, Sexuality, Race/ethnicity, U.S. citizenship, Education, Employment Status, Living as gender full-time, Outness, Support from family, co-workers or classmates, State, Racial/ethnic minority	Bivariate analyses and multilevel multivariate logistic regression
Hill et al. (2018)	United States	data collected through TLDEF Name Chance Project	Trans women of color (18–35)	65	Age	Bivariate associations and binary logistic regression
Jaffee et al. (2016)	United States	National Transgender Discrimination Survey	Transgender adults (18+)	3486	Gender spectrum, Race/ethnicity, Age, Sexuality, Education, Living as gender full-time, Income, Employment status	Bivariate associations and logistic regression
Kachen and Pharr (2020)	United States	U.S. Transgender Survey	Transgender adults (18+)	27,715 in original dataset – n after exclusion of participants not reported	Gender spectrum	Bivariate associations and multiple logistic regression
Kattari et al. (2019)	United States	Colorado Transgender Health Survey	Transgender adults (18+)	416	Gender spectrum, Sexuality, Race/Ethnicity, Education, Age	Logistic regression
Kcomt et al. (2020)	United States	U.S. Transgender Survey	Transgender adults (18+)	19,157	Gender Identity, Race/Ethnicity, Outness	Bivariate analyses and multivariate logistic regression
Lee et al. (2022)	South Korea	Rainbow Connection Project II–Korean Transgender Adults' Health Study	Transgender adults (19+)	244	Gender Spectrum	Bivariate associations and multivariable Poisson regression
Lehavot et al. (2017)	United States	data collected from veterans	Transgender veterans (18+)	298	Gender spectrum, Age, Race/Ethnicity, Relationship Status, Income	Multivariable linear regression
Lerner et al. (2021)	United States	U.S. Transgender Survey	Transgender adults (18+)	21,930	Race/ethnicity, Income, Age, Education, Citizenship	Hierarchical logistic regression
Reisner et al. (2015)^a^	United States	Data collected in Massachusetts	Transgender adults (18+)	452	Age (in years), Gender spectrum, Race/ethnicity, Income, Education, Employment status, Mode of completing survey	Multivariable logistic regression
Reisner et al. (2015)^b^	United States	National Transgender Discrimination Survey	Transmasculine adults (18+)	2578	Age range, Gender binary, Race/ethnicity, Income, Education, Sexuality, Geographic Region, Data Collection Method	Multivariable logistic regression
Seelman et al. (2017)	United States	Colorado Transgender Health Survey	Transgender adults (18+)	417	Income, Race/ethnicity	Bivariate analyses, multivariate multiple linear regression and logistic regression
Socias et al. (2014)	Argentina	data utilised from a study conducted by the Association of Transvestites, Transsexuals, and Transgenders of Argentina	Transgender women	452	Age, Place of birth, Education, Employment status (other than sex-work), Residency in Buenos Aires, Housing Stability	Bivariate analyses and multivariable logistic regression

### 2.1 Study characteristics

#### 2.1.1 Data sources

Unique datasets were used by only nine studies [[18], [19], [20], [21], [22], [23], [24], [25], [26]]. The remaining 10 studies used data from three secondary datasets: the National Transgender Discrimination Survey (NTDS) [13,[27], [28], [29]], the U.S. Transgender Survey (USTS) [[30], [31], [32], [33]], and the Colorado Transgender Health Study (CTHS) [3,34].

#### 2.1.2 Study participants

The size of study samples varied widely, (65–27,715), with a median of 1350 (IQR = 11,437).^1^ Most samples were general, comprised of transgender people over a certain age. However, three studies used samples of trans women/trans-feminine people exclusively [22,26,27], one further narrowing to trans women of color [22], while one study used a sample of trans men/trans-masculine people exclusively [29]. Two further studies sampled trans people with additional shared characteristics: black trans youth [21] and trans veterans [24].

#### 2.1.3 Quality assessment

Quality assessment using the JBI was marked on a scale of one to seven. Overall, quality of studies was moderate to high (*Mdn* *=* *5, IQR* = 0). A common issue among studies was a lack of validated and reliable variable measurements, as seen in Table 4. However, other issues addressed in the JBI checklist were uncommon.

**Table 4:** Quality Assessment using the JBI Checklist for Analytical Cross-Sectional Studies.

Study	Score (Range: 1–7)	Were the criteria for inclusion in the sample clearly defined?	Were the study subjects and the setting described in detail?	Was the exposure measured in a valid and reliable way?	Were confounding factors identified?	Were strategies to deal with confounding factors stated?	Were the outcomes measured in a valid and reliable way?	Was appropriate statistical analysis used?
Aboussouan et al. (2019)	2	Yes	Unclear	No	No	No	No	Yes
Bauer et al. (2014)	3	Yes	Yes	No	No	No	No	Yes
Cruz (2014)	5	Yes	Yes	No	Yes	Yes	No	Yes
Feldman et al. (2021)	5	Yes	Yes	Unclear	Yes	Yes	No	Yes
Glick et al. (2018)	5	Yes	Yes	No	Yes	Yes	No	Yes
Goldenberg et al. (2019)	5	Yes	Yes	No	Yes	Yes	No	Yes
Goldenberg et al. (2020)	6	Yes	Yes	Yes	Yes	Yes	No	Yes
Hill et al. (2018)	6	Yes	Yes	Yes	Yes	Yes	Unclear	Yes
Jaffee et al. (2016)	5	Yes	Yes	No	Yes	Yes	No	Yes
Kachen and Pharr (2020)	5	Yes	Yes	No	Yes	Yes	No	Yes
Kattari et al. (2019)	5	Yes	Yes	No	Yes	Yes	No	Yes
Kcomt et al. (2020)	5	Yes	Yes	No	Yes	Yes	No	Yes
Lee et al. (2022)	5	Yes	Yes	No	Yes	Yes	No	Yes
Lehavot et al. (2017)	5	Yes	Yes	Unclear	Yes	Yes	No	Yes
Lerner et al. (2021)	5	Yes	Yes	Unclear	Yes	Yes	No	Yes
Reisner et al. (2015)^a^	5	Yes	Yes	Unclear	Yes	Yes	No	Yes
Reisner et al. (2015)^b^	5	Yes	Yes	No	Yes	Yes	No	Yes
Seelman et al. (2017)	6	Yes	Yes	No	Yes	Yes	Yes	Yes
Socias et al. (2014)	4	No	Yes	No	Yes	Yes	No	Yes

#### 2.1.4 Operationalization of HAD

All studies measured HAD as one or multiple binomial variables with yes/no responses. However, there was notable variation in measurement, as shown in Table 3. Studies varied in whether they measured HAD in point or lifetime prevalence, with 11 studies measuring HAD in point prevalence of 12 months [3,20,[23], [24], [25],[30], [31], [32], [33], [34]] or six months [21], while the remaining eight studies stated or implied lifetime prevalence.

Four studies reported on HAD without an attributable reason in their measurement [21,[23], [24], [25]], while most studies asked about HAD in the context of specific reasons, most commonly discrimination [3,13,24,[27], [28], [29], [30], [31], [32], [33], [34]], cost [13,20,22,24,31], and transgender identity or expression [19,22,26].

Studies also varied in contexts and settings of HAD, where most studies measured HAD of healthcare needed when the participant is sick or injured [3,13,22,24,25,28,29,32,33], preventative healthcare [21,25,27,29], or general healthcare settings [18,20,23,26,30,31,34].

### 2.2 Factors explored in association with HAD

Included studies investigated a range of demographic variables, which are divided into transgender-specific demographics, and general demographics. Table 5 shows details of all variables studied in relation to HAD.

**Table 5:** Statistical information for all associations.

	Study	Analytic Test	Variable tested in Association with HAD	Findings				
Sex, Gender and Identity	Aboussouan et al. (2019)	Bivariate associations	Sex Assigned at Birth	Non-veterans: x2 = 20.00(1),p < .001		Veterans: x2 = 0.79(1),p > .05		
			Gender Binary	Non-veterans: x2 = 2.44(1),p > .05		Veterans: x2 = 0.66(1),*p* > .05)		
	Bauer et al. (2014)	Bivariate associations	Gender Spectrum	x2 = 3.58(1),p > .05				
	Cruz (2014)⁎	Binary logistic regression and multinomial logistic regression	Gender Spectrum	MtX: General HAD: OR = 0.484,*p* < .001 HAD due to affordability: OR = 0.8, *p* > .05 HAD due to discrimination: OR = 0.0648,p < .01		FtM: General HAD: OR = 1.406,*p* < .001 HAD due to affordability: OR = 1.116,p > .05 HAD due to discrimination: OR = 1.414,p < .01	FtX: General HAD: OR = 1.534, p < .01 HAD due to affordability: OR = 1.133, *p* > .05 HAD due to discrimination: OR = 1.564, *p* < .01	
				Referent category: MtF				
			Identifying with transsexual identity	Somewhat: On entry of variable in logistic regression: OR = 1.454,p < .001 At final step of logistic regression model: OR = 1.187, p > .05 HAD due to affordability: OR = 1.266, p > .05 HAD due to discrimination: OR = 1.14, p > .05		Strongly: On entry of variable in logistic regression: OR = 1.388,p < .001 At final step of logistic regression model: OR = 1.064, p > .05 HAD due to affordability: OR = 1.188, p > .05 HAD due to discrimination: OR = 1.033, p > .05		
			Identifying with genderqueer Identity	Somewhat: General HAD: OR = 1.314, *p* < .01; HAD due to affordability: OR = 1.286, *p* < .05; HAD due to discrimination: OR = 1.358, p < .01		Strongly: General HAD: OR = 1.472, p < .001; HAD due to affordability: OR = 1.401, p < .01; HAD due to discrimination: OR = 1.542, p < .001		
	Feldman et al. (2021)	Logistic regression	Gender Spectrum	Transgender men: aOR = 1.59, 95% CI (0.60–4.20), *p* > .05		Nonbinary: aOR = 2.30, 95% CI (0.95–5.55), *p* > .05		
				Referent category: transgender women				
				Nonbinary: aOR = 3.66, 95% CI (1.45–9.26), *p* < .01, Referent category: transgender men				
	Goldenberg et al. (2019)	Bivariate associations and multivariate logistic regression	Gender Spectrum	Transmasculine: OR = 2.11, 95% CI (0.43–10.49), *p* = .360 Gender diverse: OR = 1.92, 95% CI (0.56–6.56), *p* = .298 Referent category: transfeminine Bivariate analysis: x2 = 8.41, *p* = .015, phi = 0.28				
	Goldenberg et al. (2020)	Multilevel multivariate logistic regression	Gender Spectrum	Transmasculine: aOR = 1.63, 95% CI (1.49–1.78), p < .001 Gender diverse: aOR = 0.60, 95% CI (0.51–0.71), p < .001 Referent category: transfeminine Gender diverse: aOR = 0.83, 95% CI (0.75–0.93), *p* = .001 Referent category: transmasculine				
	Jaffee et al. (2016)	Bivariate associations	Gender Spectrum	Transgender men (41%) compared to transgender women (22%), x2 = 150.99, *p* < .001, phi = 0.21				
	Kachen and Pharr (2020)	Multiple logistic regression	Gender Spectrum	Transgender men: HAD due to cost: aOR = 1.15, 95% CI (1.05–1.25), *p* < .05; HAD due to discrimination: aOR = 1.06, 95% CI (0.96–1.16), *p* > .05		Nonbinary: HAD due to cost: aOR = 1.42, 95% CI (1.29–1.57), p < .05; HAD due to discrimination: aOR = 0.58, 95% CI (0.51–0.65), p < .05		
				Referent category: transgender women				
	Kattari et al. (2019)	Logistic regression	Gender Spectrum	Transmasculine: aOR = 1.94, 95% CI (1.01–3.73), p < .05		Nonbinary: aOR = 0.75, 95% CI (0.33, 1.71), *p* < .01		
				Referent category: transfeminine				
	Kcomt et al. (2020)	Multivariate logistic regression	Gender Spectrum	Transgender men: aOR = 1.32, 95% CI (1.21–1.45), p < .001		Nonbinary/Genderqueer: aOR = 0.71, 95% CI (0.63–0.80), *p* < .001	Cross-dressers: aOR = 0.66, 95% CI (0.53–0.82), p < .001	
				Referent category: transgender women				
	Lee et al. (2022)	Bivariate associations	Gender Spectrum	x2 = 7.48, p = .024, phi = 0.175				
	Lehavot et al. (2017)	Multivariable linear regression	Gender Spectrum	Transgender men: HAD of medical care: RR = 1.01, 95% CI (0.57–1.82), *p* > .05; HAD of mental healthcare: RR = 0.67, 95% CI (0.34–1.33), p > .05 Referent category: transgender women				
	Reisner et al. (2015)^a^	Multivariable logistic regression	Gender Spectrum	Transgender men: HAD when sick/injured: aRR = 3.15, 95% CI (2.25–4.41), *p* < .0001; HAD of routine preventative care: aRR = 2.05, 95% CI (1.55–2.72), p < .0001; HAD resulting in emergency: aRR = 1.72, 95% CI (1.18–2.49), *p* = .005 Referent category: transgender women				
	Reisner et al. (2015)^b^	Multivariable logistic regression	Gender Binary	Binary identity: HAD when sick/injured: RR = 1.37, 95% CI (1.07–1.76), *p* = .012; HAD of preventative care: RR = 1.18, 95% CI (0.93–1.49), *p* = .172 Referent category: nonbinary				
Social Transition Factors	Goldenberg et al. (2020)	Bivariate associations and multilevel multivariate logistic regression	Level of Outness	aOR = 1.00, (0.98–1.03), *p* = .614; significant in bivariate analysis, *p* > .001				
			Living as Gender Full-Time	aOR = 1.86, 95% CI (1.70–2.04), *p* < .001				
			Perceiving support from family, co-workers, or classmates	aOR = 0.81, 95% CI (0.75–0.88), <0.001				
	Jaffee et al. (2016)†	Logistic regression	Living as Gender Full-time	Transgender men: OR = 1.02, 95% CI (0.71–1.46)		Transgender women: OR = 0.94, 95% CI (0.70–1.28)		
	Kcomt et al. (2020)	Multivariate logistic regression	Disclosed to Everyone in their Social Network	aOR = 0.77, 95% CI (0.68–0.87), *p* < .05				
Age	Aboussouan et al. (2019)	Bivariate associations	Age in years	Veterans: HAD (M = 40.48, SD = 13.39), No HAD (M = 46.45,SD = 14.30), t(151) = −2.50, *p* = .014		Non-veterans: HAD (M = 27.55, SD = 10.08), No HAD (M = 29.29, SD = 10.68, t(2780.10) = −4.45,*p* < .001)		
	Cruz (2014)⁎	Bivariate associations	Age in years	General HAD: OR = 0.987, *p* < .001 HAD due to affordability: OR = 0.99, *p* > .05 HAD due to discrimination: OR = 0.977, p > .05				
	Glick et al. (2018)	Bivariate associations and multivariate logistic regression	Age (18–24, 25–44, 45–54, 55+)	x2 = 52.22, *p* < .001, v = 0.154 significant in multivariate analysis (statistical data not shown)				
	Goldenberg et al. (2019)	Multivariate logistic regression	Age in years	OR = 1.10, 95% CI [0.86, 1.40], *p* = .455				
	Goldenberg et al. (2020)	Multilevel multivariate logistic regression	Age in years	aOR = 0.98, [0.98–0.99], *p* < .001				
	Jaffee et al. (2016)†	Logistic regression	Age (18–24, 25–44, 45+)	25–44: Transgender men: aOR = 1.06, 95% CI (0.80–1.40) Transgender women: aOR = 1.05, 95% CI (0.72–1.53) Referent category: 18–24		45+: Transgender men: aOR = 0.51; 95% CI (0.32–0.83) Transgender women: aOR = 0.58; 95% CI (0.39–0.87)		
	Kattari et al. (2019)	Logistic regression	Age (<25,25–44, 45–64,65+)	25–44: aOR = 0.87, 95% CI (0.47–1.60), p > .05 Referent category: <25		45–64: aOR = 0.37, 95% CI (0.16–0.84), p < .05	65+: aOR = 0.60, 95% CI (0.11–3.27), p > .05	
	Lehavot et al. (2017)	Multivariable linear regression	Age (18–34, 35–49, 50–64, 65+)	35–49: Medical care: RR = 0.87, 95% CI (0.49–1.54), *p* > .05 Mental Healthcare: RR = 0.95, 95% CI (0.51–1.77), *p* > .05		50–64: Medical care: RR = 0.59, 95% CI (0.33–1.06), p > .05 Mental Healthcare: RR = 0.50, 95% CI 0.26–0.96), *p* < .05	65+: Medical care: RR = 0.59, 95% CI (0.26–1.30), *p* > .05 Mental Healthcare: RR = 0.30, 95% CI (0.11–0.86), p < .05	
				Referent category: 18–34 Significant in bivariate analysis (*p* < .05)				
	Lerner et al. (2021)	Hierarchical logistic regression	Age in years	OR = 1.00, 95% CI (1.00–1.00), p > .05				
	Reisner et al. (2015)^a^	Multivariable logistic regression	Age in years	Care resulting in emergency: RR = 1.00, 95% CI (0.99–1.01), *p* = .667 Care when sick/injured: RR = 0.98, 95% CI (0.97–0.99), *p* = .010 Preventative care: RR = 0.98, 95% CI (0.97–0.99), *p* < .0001				
	Reisner et al. (2015)^b^	Multivariable logistic regression	Age (18–24, 25–44, 45+)	18–24: Preventative care: RR = 0.73, 95% CI (0.59–0.91), *p* = .005 Care when sick/injured: RR = 0.81, 95% CI (0.65–1.01), *p* = .060 Referent category: 25–44		45+ Preventative care: RR = 0.58, 95% CI (0.42–0.81), *p* = .001 Care when sick/injured: RR = 0.58, 95% CI (0.40–0.83), *p* = .003		
	Socias et al. (2014)	Bivariate associations	Age in years	Bivariate: OR = 1.02, 95% CI (0.70–148), *p* > .10 Not entered in multivariate analysis				
Race/Ethnicity	Aboussouan et al. (2019)	Bivariate associations	Part of a racial minority	Veterans: x2 = 2.15 (1), p > .05		Nonveterans: x2 = 4.66 (1), *p* < .05 OR = 1.22, 95% CI (1.02,1.45)		
	Cruz (2014)⁎	Binary logistic regression and multinomial logistic regression	Race/Ethnicity (White, Black, Asian, Latinx, Native American, Multiracial)	Black: General HAD: OR = 0.864, *p* > .05 HAD due to affordability: OR = 0.665, p > .05 HAD due to discrimination: OR = 1.099, p > .05	Asian: General HAD: OR = 0.512, *p* < .01 HAD due to affordability: OR = 0.453, *p* < .05 HAD due to discrimination: OR = 0.57, p > .05	Latinx: General HAD: OR = 0.761, p > .05 HAD due to affordability: OR = 0.519, p < .05 HAD due to discrimination: OR = 0.988, p > .05	Native American: General HAD: OR = 1.679, p > .05 HAD due to affordability: OR = 1.438, p > .05 HAD due to discrimination: OR = 1.961, p > .05	Multiracial: General HAD: OR = 1.41, p < .01 HAD due to affordability: OR = 1.155, p > .05 HAD due to discrimination: OR = 1.663, p > .05
				Referent category: White				
	Glick et al. (2018)	Bivariate associations	Person of Color	x2 = 18.55, p < .001, phi = 0.092				
	Goldenberg et al. (2020)	Multilevel multivariate logistic regression	Race/Ethnicity (Native American, Asian and Pacific Islander, Black, Latinx/Hispanic, Multiracial, White, other	Native American: aOR = 1.32, 95% CI (0.99–1.77), *p* = .059 Asian and Pacific Islander: aOR = 0.93, 95% CI (0.75–1.16), *p* = .511 Black: aOR = 0.95, 95% CI (0.77–1.18), *p* = .661) Latinx/Hispanic: aOR = 0.97, 95% CI (0.82–1.14), *p* = .686 Multiracial: aOR = 1.01, 95% CI (0.86–1.20), *p* = .842 other: aOR = 0.96, 95% CI (0.74–1.25), *p* = .749				
				Referent category: White Significant in bivariate analysis, *p* < .001				
	Jaffee et al. (2016)†	Logistic regression	Race/Ethnicity (White, Black, Asian, Latinx, Native American, Multiracial, other)	Transgender men: Black: OR = 1.43, 95% CI (0.85–2.42) Latinx: OR = 1.45, 95% CI (0.91–2.31) Native American: OR = 2.12, 95% CI (1.13–3.97), significant Multiracial: OR = 1.99, 95% CI (1.25–3.16), significant Other: OR = 0.86, 95% CI (0.43–1.72)		Transgender women: Black: OR = 0.89, 95% CI (0.53–1.50) Latinx: OR = 1.63, 95% CI (0.95–2.80) Native American: OR = 1.45, 95% CI (0.89–2.35) Multiracial: OR = 1.69, 95% CI (1.06–2.69), significant Other: OR = 0.84, 95% CI (0.37–1.90)		
				Referent category: White				
	Kattari et al. (2019)	Logistic regression	Person of Color	OR = 1.65 95% CI (0.86–3.15), p > .05				
	Kcomt et al. (2020)	Multivariate logistic regression	Race/Ethnicity (Black/African American, Latinx/Hispanic, Biracial/Multiracial, Non-Hispanic White, others)	Black/ African American: aOR = 1.11, 95% CI (1.00–1.24), p > .05	Latinx/Hispanic: aOR = 1.49, 95% CI (1.34–1.65), *p* < .001	Biracial/Multiracial: aOR = 1.32, 95% CI (1.03–1.69), *p* < .05	Others: aOR = 1.49, 95% CI (1.24–1.78), p < .001	
				Referent Category: Non-Hispanic White				
	Lehavot et al. (2017)	Multivariable linear regression	Nonwhite race/ethnicity	Medical care: RR = 0.89, 95% CI (0.49–1.64), p > .05 Mental Healthcare: RR = 0.82, 95% CI (0.41–1.65), p > .05				
	Lerner et al. (2021)	Hierarchical logistic regression	Person of Color	OR = 1.24, 95% CI (1.13–1.37), p < .001				
	Reisner et al. (2015)^a^	Multivariable logistic regression	Person of Color	Resulting in emergency care: aRR = 0.41, 95% CI (0.27–0.64), *p* <. 0001 Care when sick/injured: aRR = 0.27, 95% CI (0.18–0.40), *p* < .0001 Preventative care: aRR = 0.42, 95% CI (0.30–0.58), p < .0001				
	Reisner et al. (2015)^b^	Multivariable logistic regression	Person of Color	Care when sick or injured: RR = 1.29, 95% CI (1.04–1.60), *p* = .023) Preventative care: RR = 1.19, 95% CI (0.97–1.46), *p* = .089				
	Seelman et al. (2017)	Bivariate associations	Person of Color	x2 = 3.65, *p* < .10, phi = 0.102				
Socioeconomic Factors	Cruz (2014)⁎	Binary logistic regression and multinomial logistic regression	Income (<20 k, 20-40 k, 40-60 k, 60-100 k, 100 k+)	20-40 k: General HAD: OR = 1.056, *p* > .05 HAD due to affordability: OR = 1.213, *p* > .05 HAD due to discrimination: OR = 0.884, *p* > .05	40-60 k: General HAD: OR = 653, *p* < .001 HAD due to affordability: OR = 0.631, p < .001 HAD due to discrimination: OR = 0.664, *p* < .01	60-100 k: General HAD: OR = 454, p < .001 HAD due to affordability: OR = 0.437, p < .001 HAD due to discrimination: OR = 0.467, p < .001	100 k+ General HAD: OR = 0.378, p < .001 HAD due to affordability: OR = 0.251, p < .001 HAD due to discrimination: OR = 0.512, p < .001	
				Referent Category: <20 k				
	Glick et al. (2018)	Bivariate analyses and multivariate logistic regression	Income (<20 k, 20-50 k, 50 k+)	x2 = 56.03, p < .001, v = 0.159 significant in multivariate analysis: findings not shown				
	Goldenberg et al. (2020)	Multilevel multivariate logistic regression	Employment status (Employed, unemployed, out of the labor force)	Unemployed: aOR = 0.81, 95% CI (0.73–0.89), *p* < .001		Out of the labor force: aOR = 0.85, 95% CI (0.78–0.93), p < .001		
				Referent Category: Employed				
	Jaffee et al. (2016)†	Logistic regression	Income (<20 k, 20-60 k, 60 k+)	Transgender Men: 20-60 k: aOR = 1.00, 95% CI (0.75–1.33) 60 k: aOR = 0.84, 95% CI (0.60–1.19)		Transgender Women: 20-60 k: aOR = 0.72, 95% CI (0.53–0.97), significant 60 k+: aOR = 0.56, 95% CI (0.39–0.80)		
				Referent category: <20 k				
			Employed	Transgender Men: aOR = 0.67, 95% CI (0.50–0.90)		Transgender Women: aOR = 1.17, 95% CI (0.89–1.55)		
	Lehavot et al. (2017)	Multivariable linear regression	Income of less than 35 k	Medical care: RR = 1.14, 95% CI (0.72–1.81), *p* > .05; significant at bivariate analysis, *p* < .05 Mental Healthcare: RR = 0.93, 95% CI (550–1.56), *p* > .05				
	Lerner et al. (2021)	Hierarchical logistic regression	Income of more than 20 k	OR = 1.05, 95% CI (0.96–1.15), p > .05				
	Reisner et al. (2015)^a^	Multivariable logistic regression	Income of less than 20 k	Resulting in emergency care: aRR = 1.38, 95% CI (1.00–1.92), p < .05 Care when sick/injured: aRR = 1.47, 95% CI (1.12–1.93), p < .05 Preventative care: aRR = 1.81, 95% CI (1.42–2.310, *p* < .0001				
			Employed	Resulting in emergency care: aRR = 1.09, 95% CI (0.71–1.67) *p* = .160 Care when sick/injured: aRR = 0.91, 95% CI (0.69–1.21), *p* = .528 Preventative care: aRR = 1.40, 95% CI (1.08–1.80), p = .010				
	Reisner et al. (2015)^b^	Multivariable logistic regression	Income (<20 k, 20-49 k, 50-99 k)	<20 k: Care when sick or injured: RR = 1.53, 95% CI (1.20–1.97), *p* = .0007) Preventative care: RR = 1.57, 95% CI (1.23–2.00), *p* = .0003		20-49 k: Care when sick or injured: RR = 1.18, 95% CI (0.94–1.48), p = .160 Preventative care: RR = 1.26, 95% CI (1.01–1.56, *p* = .040)		
				Referent Category: 50-99 k				
	Seelman et al. (2017)	Bivariate associations	Income	x2 = 12.18, p < .01, v = 0.187				
	Socias et al. (2014)	Bivariate associations and multivariable logistic regression	Employed (Other than sex work)	aOR = 1.00, 95% CI (0.56–1.79), *p* > .05 significant at bivariate analysis: OR = 0.63, 95%CI (0.56–1.79) p < .05				
		Bivariate associations	Housing Stability	Bivariate: OR = 0.92, 95% CI (0.58–1.45), p > .05 Not entered in multivariate analysis				
Veteran Status	Aboussouan et al. (2019)	Bivariate associations	Veteran Status	x2 = 7.73 (1), *p* < .01, phi = 0.05				
Education Level	Glick et al. (2018)	Multivariate logistic regression	Education level (High school or less, Some college, College degree, Graduate degree)	x2 = 11.38, *p* < .05, v = 0.072				
	Goldenberg et al. (2019)	Multivariate logistic regression	High school education	OR = 1.55, 95% CI (0.34–7.14), *p* = .572				
	Goldenberg et al. (2020)	Multilevel multivariate logistic regression	Education level (Less than high school, High school graduate, Some college, Undergraduate degree, Graduate or professional degree)	High school graduate: aOR = 1.20, 95% CI (0.97–1.49), p = .089	Some college: aOR = 1.32, 95% CI (1.08–1.61), *p* = .007	Undergraduate degree: aOR = 1.64, (1.34–2.02), *p* < .001	Graduate degree: aOR = 1.76, 95% CI (1.40–2.21), p < .001	
				Referent category: Less than high school				
	Jaffee et al. (2016) †	Logistic regression	Education level (High school or less, Some college, College degree, Graduate degree)	Transgender men: Some college: aOR = 1.57, 95% CI (0.97–2.55) College Degree: aOR = 1.34, 95% CI (0.82–2.20) Graduate Degree: aOR = 2.13, 95% CI (1.27–3.58) Referent category: High school or less		Transgender women: Some college: aOR = 1.08, 95% CI (0.74–1.58) College Degree: aOR = 0.86, 95% CI (0.56–1.34) Graduate Degree: aOR = 1.21, 95% CI (0.77–1.90)		
	Kattari et al. (2019)	Logistic regression	Education level (Some high school/GED, Some college, Bachelors, Graduate degree)	Some college: aOR = 2.16, 95% CI (0.89–5.25), p < .05		Bachelors: aOR = 1.80, 95% CI (0.68–4.78), p < .05	Graduate degree: aOR = 1.56, 95% CI (0.53–4.63), p < .05	
				Referent category: Some high school/GED				
	Lerner et al. (2021)	Hierarchical logistic regression	More than high school education	OR = 1.24, 95% CI (1.14–1.35), p < .001				
	Reisner et al. (2015)^a^	Multivariable logistic regression	Low education	Care resulting in emergency: RR = 1.09, 95% CI (0.71–1.67), *p* = .698 Care when sick/injured: RR = 0.51, 95% CI (0.33–0.78), *p* = .002 Preventative care: RR = 0.71, 95% CI (0.49–1.04), *p* < .08				
	Reisner et al. (2015)^b^	Multivariable logistic regression	Education level (High school or less, Some college, College degree, Graduate degree)	High school or less: Preventative care: RR = 0.66, 95% CI (0.47–0.95), *p* = .024 Care when sick/injured: RR = 0.68, 95% CI (0.48–0.98), *p* = .039	College degree: Preventative care: RR = 0.66, 95% CI (0.47–0.95), p = .024 Care when sick/injured: RR = 0.68, 95% CI (0.48–0.98), p = .039		Graduate degree: Preventative care: RR = 1.75, 95% CI (1.36–2.26), p < .0001 Care when sick/injured: RR = 1.52, 95% CI (1.17–1.97), p = .002	
				Referent category: Some college				
	Socias et al. (2014)	Bivariate associations	High school education or higher	OR = 0.72, 95% CI (0.46–1.64), p > .10 Not entered in multivariate analysis				
Sexuality	Goldenberg et al. (2020)	Multivariate logistic regression	Sexuality (Heterosexual, LGB, Asexual, other)	LGB: aOR = 1.06, 95% CI (0.95–1.20), *p* = .301	Asexual: aOR = 1.05, 95% CI (0.90–1.23), *p* = .524		Other: aOR = 1.10, 95% CI (0.92–1.30), *p* = .292	
				Referent category: Heterosexual Significant in bivariate analysis, p < .001				
	Jaffee et al. (2016)†	Logistic regression	Sexuality (Heterosexual, Queer, LGB, Asexual/other)	Queer: Transgender men: aOR = 1.55, 95% CI (1.13–2.13) Transgender women: aOR = 1.28, 95% CI (0.76–2.18)	LGB: Transgender men: aOR = 1.00, 95% CI (0.71–1.41) Transgender women: aOR = 0.84, 95% CI (0.63–1.13)		Asexual/other: Transgender men: aOR = 1.21, 95% CI (0.78–1.89) Transgender women: aOR = 0.95, 95% CI (0.66–1.37)	
				Referent category: Heterosexual				
	Kattari et al. (2019)	Logistic regression	Sexuality (Heterosexual, LGSGL, queer, bisexual/pansexual, Questioning/not sure)	LGSGL: aOR = 1.77, 95% CI (0.68–4.59), p > .05	Queer: aOR = 4.19, 95% CI (1.65, 10.62), p < .01	Bisexual/pansexual: aOR = 1.57, 95% CI (0.63–3.93), p > .05	Questioning: aOR = 0.62, 95% CI (0.12–3.29), p > .05	
				Referent category: Heterosexual				
	Reisner et al. (2015)^b^	Multivariable logistic regression	Sexuality (Heterosexual, Gay, Bisexual, Queer)	Heterosexual: Preventative care: RR = 0.54, 95% CI (0.41–0.71), p < .0001 Care when sick/injured: RR = 0.63, 95% CI (0.48–0.82), *p* = .0008	Gay: Preventative care: RR = 0.60, 95% CI (0.47–0.76), p < .0001 Care when sick/injured: RR = 0.73, 95% CI (0.57–0.95), *p* = .018		Bisexual: Preventative care: RR = 0.52, 95% CI (0.39–0.70), p < .0001 Care when sick/injured: 0.71, 95% CI (0.53–0.96), *p* = .027	
				Referent Category: Queer				
Relationship Status	Cruz (2014)⁎	Binary logistic regression and multinomial logistic regression	Relationship status (Single, Partnered, Married/Civil union, Separated/divorced/widowed)	Partnered: General HAD: OR = 1.61, p < .001 HAD due to affordability: OR = 1.728, p < .001 HAD due to discrimination: OR = 1.512, p > .05	Married/Civil union: General HAD: OR = 1.497, p < .001 HAD due to affordability: OR = 1.631, p < .001 HAD due to discrimination: OR = 1.401, p < .01		Separated/divorced/widowed: General HAD: OR = 1.294, p < .05 HAD due to affordability: OR = 1.539, p < .01 HAD due to discrimination: OR = 1.019, p > .05	
				Referent category: Single				
	Glick et al. (2018)	Bivariate associations	Relationship status (Single, Partnered, Married/Civil union, Separated/divorced/widowed)	x2 = 15.56, p < .01, v = 0.083				
	Lehavot et al. (2017)	Multivariable linear regression	Unmarried/No domestic partner	medical care: RR = 0.93, 95% CI (0.60–1.43), p > .05 mental health care: RR = 0.96, 95% CI (0.60–1.55), p > .05				
Location-Based Demographics	Goldenberg et al. (2020)	Multilevel multivariate logistic regression	U.S. Citizenship	aOR = 0.96, 95% CI (0.74–1.25), *p* = .749				
			State proportion of non-Hispanic white people	aOR = 0.99, 95% CI (0.99–1.00), *p* = .068				
			State population density	aOR = 1.00, 95% CI (1.00–1.00), *p* = .833				
			State proportion living in a rural area	aOR = 1.22, 95% CI (0.07–22.64), *p* = .892				
			State proportion living in an urban area	aOR = 0.94, 95% CI (0.72–1.22), *p* = .636				
	Lerner et al. (2021)	Hierarchical logistic regression	U.S. Citizenship	OR = 1.05, 95% CI (0.91–1.07), p > .05				
	Reisner et al. (2015)^b^	Multivariable logistic regression	U.S. Geographic Region (California, Midwest/West, New England/New Atlantic, Southern)	California: Preventative care: RR = 1.63, 95% CI (1.23–2.15), p = .0008 Care when sick/injured: RR = 1.48, 95% CI (1.12–1.95), *p* = .005	New England/Mid Atlantic: Preventative care: RR = 1.00, 95% CI (0.81–1.24), *p* = .995 Care when sick/injured: RR = 0.98, 95% CI (0.79–1.22), *p* = .880		Southern: Preventative care: RR = 1.14, 95% CI (0.87–1.49), *p* = .329 Care when sick/injured: RR = 1.05, 95% CI (0.79–1.40), *p* = .743	
				Referent category: Midwest/West				
	Socias et al. (2014)	Bivariate associations	Born outside Argentina	OR = 0.87 [0.46–1.48], *p* > 10 Not entered in multivariate analysis				
		Multivariable logistic regression	Residency in Buenos Aires metropolitan area	aOR = 2.32, 95% CI (1.44–3.76), p < .05				

Unless stated otherwise, OR, RR and PR referent categories denote the absence of exposure to the variable.

Abbreviations: HAD = Healthcare avoidance & delay, LGB = lesbian, gay and bisexual, LGSGL = Lesbian, gay, same gender loving.

⁎ Insufficient data to calculate confidence intervals.

† *P* values unreported.

#### 2.2.1 Transgender-specific demographics

**Sex and Gender.** Sex and gender identification were investigated in relation to HAD by 14 studies, with data drawn from 10 unique datasets [13,[18], [19], [20], [21],[23], [24], [25],[28], [29], [30], [31], [32],^34^].

Thirteen of these studies investigated HAD in relation to gender spectrum or biological sex, measuring either differences between gender identity or birth sex, with ten studies contributing to evidence that transgender men or trans-masculine people engage in HAD more than transgender women or trans-feminine people [13,18,21,23,25,28,[30], [31], [32],^34^].

Eight of these studies found that transmasculine people had between 15% to 95% higher likelihood or risk of HAD as transfeminine people. One study found notably higher risk of postponement for preventative care (aRR = 2.05) and for care when sick/injured (aRR = 3.15) [25]. Another study similarly found over twice the likelihood of HAD for transgender men, though this effect was not found to be significant in multivariate analysis [21]. There was some conflict in results by reason for HAD, with a significant gender effect for HAD due to cost, but not for HAD due to discrimination in one study [31], while another found that gender was predictive of variation in HAD due to discrimination and not HAD due to cost [13].

Nine studies further investigated HAD with separate categories or variables relating to specific nonbinary, gender diverse, or genderqueer identities [13,18,20,21,[29], [30], [31], [32],^34^]. Overall, results were mixed, as four studies found evidence to suggest that HAD was lower in nonbinary people, comparing them to samples of trans women [32,34], trans men [29] or both [30], while two studies found evidence of higher HAD in those with nonbinary identities [13,20]. However, overlap in data sources limits the strength of evidence, while partial results and variance in findings also suggests a role of additional variables and context in this association. Studies found variance in results based on assigned gender at birth [13] and reasons for HAD [13,31], while others found associations not maintained in multivariate analysis [21]. Others reported confounding effects of third variables; health insurance [32] and age [20]. One study further explored identification with the terminology ‘transsexual’, finding an increase in HAD that was rendered nonsignificant once medical transition variables were added to the model [13].

**Social Transition Factors.** Social transition factors such as general outness, social support, and living full-time as one's gender were explored cumulatively by only three studies, with limited evidence for associations [28,30,32].

Two studies, both using the USTS dataset, explored level of general outness [30], [32]. Those who disclosed their identity to everyone in their life had lower likelihood of HAD compared to those who had disclosed to less people [32], while another study found a similar effect in bivariate analysis that was not maintained in multivariate analysis [30], indicating that some of a large number of study covariates may confound this association. This study also investigated whether participants felt that they had social support, finding that having support from others was associated with less likelihood of HAD. Additionally, living as one's gender full-time was explored by two studies, with one study finding that being full-time was associated with 86% increased likelihood of avoidance [30], while the other study found no significant effect for either transgender men or women [28].

#### 2.2.2 General demographics

**Age.** Age was investigated by 12 studies [13,18,21,[24], [25], [26], [27], [28], [29], [30],^33^,^34^].

Evidence for the link between age and HAD is strong, as nine studies from six unique data sets found direct associations, generally indicating that older age was associated with lower HAD [13,18,24,[27], [28], [29],^30^,^34^]. Only one study found any effect indicating that a younger age group was associated with lower HAD, where those aged 18–24, in addition to those aged over 45, had lower risk of reporting HAD than those aged 25–44 [29]. Effect sizes were varied, ranging from very slight or small effects when age was measured as a continuous variable, to larger effects when age was treated as categorical. Studies reported between 40 and 70% lower likelihood or risk of HAD in older age groups compared to younger age groups [24,28,29,34], with effects most pronounced in highest age categories.

Two studies found variation in results for different medical contexts, one finding an effect for delaying mental health care but not for medical care, though this was significant in bivariate analysis [24], and the other finding effects for postponement of both preventative care and care when sick/injured, but no effect for care that resulted in emergency [25].

**Race/Ethnicity.** Drawing from six unique datasets, 12 studies included variables relating to race and ethnicity [3,13,18,24,25,[27], [28], [29], [30],[32], [33], [34]].

Results from eight studies comparing HAD between people of color (POC) and white counterparts reported mixed results, as four studies found evidence for increased HAD among POC [18,27,29,33]. One study found lower risk of HAD in POC [25], with two studies finding no significant difference, [24,34] and a further only significant at *p* < .10 [3]. These mixed results may be, in part, a result of variance by ethnicity, as results from four studies measuring multiple racial and ethnic categories highlight substantial variance in results by ethnicity.

From NTDS data, one study found lower HAD in Asian participants, higher HAD in Multiracial participants, and no effect for those who were Black, Native American, or Latinx [13], while the other study found higher HAD in Multiracial transgender men and women, and in Native American transgender men [28]. One of these further found that location, i.e. state of residence, mediated this association [30]. From USTS data, one study found a difference in HAD between races not maintained in multivariate analysis [30]. Effect sizes for this study indicate that most racial and ethnic minority categories demonstrated little difference in likelihood of HAD except for Native American participants, who may have higher likelihood of HAD (aOR = 1.32, 95% CI [0.99–1.77]). The other study drawing from USTS data found that Latinx, Biracial and Multiracial, and other ethnic groups had higher likelihood of HAD, with the exception of Black participants, for whom there was no difference [32].

**Socioeconomic factors.** Financial demographic factors of income, employment, and housing stability were explored by 11 studies [3,13,18,[24], [25], [26], [27], [28], [29], [30],^33^].

Income level was explored by eight studies, though drawing from five datasets [3,13,24,25,[27], [28], [29],^33^]. Findings largely indicated that higher income was associated with lower HAD [3,13,25,[27], [28], [29]], though significant findings draw from only three datasets, limiting the strength of evidence. One study also found a concurring association that was not maintained in multivariate analysis [24], indicating a possible confounding impact of demographic and health-related covariates. Findings sometimes varied for different genders, or for different income bands. One study found the association for transgender women but not for transgender men [28] while findings from studies using multiple income bands indicate more, and larger, effects the higher the income band [3,13,29]. Additionally, where associations weren't maintained in final analyses [24,33], higher income bands were set relatively low in comparison to other studies, at >20 k [33] and > 35 k [24].

Four studies investigated employment status [25,26,28,30], with mixed results. One study found an association not maintained at multivariate analysis, indicating that trans women with employment other than sex work had less likelihood of HAD [26], while another conversely found decreased likelihood of HAD for those who were unemployed or out of the labour force [30]. Similarly, in another study, being employed was associated with an increase in postponement of preventative care. However, this effect was not found for emergency care or care needed when sick/injured [25]. The remaining study found that employment was associated with lower HAD for transgender men, but no significant effect for transgender women [28]. One study investigated housing stability, finding no significant association with HAD in bivariate analysis, and further excluded it from multivariate analysis [26].

**Veteran Status.** One study investigated veteran status, finding that veterans were less likely to engage in HAD due to non-suicidal self-injury than general transgender participants [18].

**Education.** Nine studies explored levels of educational attainment in relation to HAD [21,[25], [26], [27], [28], [29], [30],^33^,^34^]. Six studies found significant effects, with results largely indicating that higher educational attainment was associated with higher HAD [25,[27], [28], [29], [30],^33^]. However, these studies collectively used only three datasets, reducing the strength of evidence, while the remaining studies each found no significant association between education and HAD. There was some variance in results, with one study finding this effect for transgender men but not for transgender women [28], while another found this effect when looking at HAD when sick or injured, but not for HAD of preventative care or HAD resulting in emergency [25].

**Sexuality and Relationship Status.** Four studies explored sexuality, though two used the same dataset [[28], [29], [30],^34^]. Results indicate some evidence for elevated HAD in sexual minority groups. Two studies found that identifying as queer was associated with much higher likelihood of HAD, compared to being heterosexual [29,34], while another study found this effect for transgender men but not for transgender women [28]. Both studies using NTDS data also found that specific identities such as gay, lesbian, bisexual or asexual did not show similar patterns, as one found that identifying as any of these identities was not associated with an increase in risk or likelihood of HAD [28], while the other found that identifying as gay or bisexual, in addition to heterosexual, had a decreased risk of HAD when compared to identifying as queer [29]. One further study found a significant bivariate difference between sexualities not maintained in multivariate analysis, indicating that the large number of covariates in the study may confound this association [30].

Three studies explored relationship status [13,24,27], two drawing from NTDS data and the other from a sample of trans veterans. Results were mixed, with limited evidence available. One NTDS study found a difference in relationship status between those who postponed care and those who didn't [27], while the other found that, compared to being single, all relationship statuses of partnered, married/in a civil union, and separated/divorced/widowed, were associated with increased likelihood of HAD [13]. The most pronounced effect was for those who were partnered, with over 60% increase in likelihood, while additional analyses indicated some minor variance according to reason for HAD, indicating that the association can mostly be explained through affordability rather than fear of discrimination. The remaining study found no significant effect [24].

**Citizenship/Place of Residence/State Demographics.** Two studies explored the relationship between HAD and U.S. citizenship, though both using the same dataset and finding no significant associations [30,33]. Similarly, an Argentinian study examined the association between foreign place of birth and HAD, finding no association [26]^.^

Two studies explored the association between place of residence and HAD. One found variance in HAD according to U.S. region [29]. Compared to those in the Midwest/West, those in California had higher risk of HAD, while there was no significant effect for those in New England/Mid Atlantic, or Southern, regions. The other study found that living in the Buenos Aires metropolitan area was associated with an increased likelihood of avoidance [26].

One study explored demographic factors of locations, looking at state population makeup [30]. Population variables of proportion of non-Hispanic white people, proportion living in a rural area, proportion living in an urban area, and population density all had no significant association with HAD.

## 3 Discussion

This review synthesizes literature exploring associations between demographic factors and HAD in the transgender population, reporting on the cumulative evidence for associations and exploring how operationalization of HAD impacts our understanding of these findings. We found consistent evidence that HAD levels varied across a range of intra-community demographic factors. Increased HAD was associated with factors such as gender identification, social transition factors, age, specific ethnic minority groups, non-normative sexuality, lower income, higher educational attainment, and living in specific locations. Consistency of evidence varied between these factors, with some consistently found to be associated with HAD, while evidence for others was hampered by sparse research and overlap between data sources.

Evidence consistently supported an association between gender identity and HAD, with transgender men and transmasculine people most likely to engage in HAD. Findings follow cisgender patterns, where men are less likely to utilize healthcare [35]. As research identifies that facets of internalized masculinity are associated with healthcare avoidance in both men and women [36], findings may indicate an internalization of gender norms in transmasculine participants. To date, limited research has addressed gender beliefs and internalization of masculinity in transmasculine individuals, and how these may impact on health decision-making. It may also be that, as transmasculine individuals have common healthcare needs, such as gynaecologic care, additive to those of transfeminine counterparts, they may have increased opportunity for HAD. Indeed, as gynaecologic care is heavily gender- and sex-coded, transgender men report a range of barriers [37], and avoid or delay use of these services at high rates [38].

Findings also highlight gaps in knowledge on intra-community gender differences in HAD. Evidence for association between HAD and gender identities outside of the male-female binary was mixed, with partial effects and variance in findings. As most research addresses binary transgender experiences, and research indicates that nonbinary people face specific forms of invalidation in healthcare settings and use healthcare avoidance as a management strategy [39], more research is necessary to further disentangle how nonbinary or gender variant people may experience HAD. Conflicting evidence also emerged for gender differences between HAD attributable to cost and HAD attributable to discrimination, indicating variation in associations with HAD for different reasons, and highlighting the need for further research to understand the role that contextual factors may play in this relationship.

There was little research investigating other transgender-specific demographic factors. Social transition factors such as living as one's gender full-time, disclosure of transgender identity, and social support were found to be associated with HAD, though conclusions are drawn from a very small body of research. While findings are limited, alongside previous review findings that name and gender change may also be associated with decreased HAD [9], they highlight the importance of further research exploring how social transition factors may relate to HAD.

Like gender identification, younger age emerged as commonlyassociated with increased HAD, with evidence coming from a wide range of studies and datasets. This is consistent with findings from cisgender samples [40], and may be an indication of increased medical need for older transgender adults. Indeed, personal assessment of need has been identified as a core contributor to health service utilization in influential models such as the Behavioral Model (BM) [41]. Seen through the theoretical lens of the Health Belief Model (HBM) [42], older transgender adults may have similar perceived barriers to engaging with healthcare services as younger adults, but are likely to have higher perceived susceptibility and severity of illness, and higher perceived benefits of engaging with healthcare services as they age. This may, in part, explain differences in utilization in older cohorts. Additionally, older transgender adults may have access to better care due to increased financial resources. While findings indicate that younger transgender adults are particularly at risk, however, older transgender adults may also be at elevated risk in comparison with cisgender counterparts.

Increased HAD was also sometimes found in participants from specific racial and ethnic groups; those who were Multiracial, Latinx, or Native American. Increased levels of HAD in POC is consistent with research indicating that marginalized groups are more likely to engage in avoidance, both generally and due to discrimination [43]. However, black participants were not found to engage in higher HAD than white counterparts in any studies, while Asian participants were found to engage in HAD less than white counterparts in one study, which would provide evidence counter to this explanation. However, the body of evidence in this area does not allow for clear conclusions, as only a small number of studies drawing from two secondary datasets investigated differences between multiple racial and ethnic groups.

More common was the investigation of differences in HAD between two racial groups, white non-Hispanic people and POC. Findings in this area were again mixed, which may be due, in part, to differences in the racial and ethnic makeup of samples between studies. Findings show a substantial gap in the literature addressing how living with multiple forms of marginalization may interact and relate to HAD in the transgender population. Considering intersections of marginalization is vital to our understanding of how power relations may contribute to health inequity in the trans population [14]. As transgender POC experience marginalization and discrimination in healthcare both as a result of their gender and their races [44], further research implementing an intersectional approach on HAD would contribute much to unpacking the nature of this relationship, and also would disentangle differences in HAD between trans people of different racial and ethnic backgrounds.

Such an approach would also benefit our understanding of the relationship between sexuality and HAD, as findings indicate some tentativeevidence for association with sexual minority statusThis may in part be explained, as with race and ethnicity, by processes of multiple marginalization, and should be further investigated in future research. Interestingly, however, significant associations with increased HAD were largely confined to identification with the label “queer”, not specific sexual identities of gay, lesbian, bisexual, asexual, etc. This association may thus also overlap with other demographic factors such as age, as younger people are much more likely to identify with the queer label than older gender and sexual minority people [45].

Lower income level was also associated with increased HAD. Though housing stability was not associated with HAD, the majority of findings do indicate the importance of socioeconomic factors in HAD. This can be considered alongside findings from a previous review, which highlight the importance of the relationship between health insurance status and HAD [46]. As transgender individuals are more likely to experience a variety of financial hardships [47], the cost of accessing healthcare services is of key importance in understanding patterns of utilization and HAD in this population. Notably, a comparison of study results differing in operationalization of HAD indicates that level of income was associated with not only HAD due to cost, but also HAD more generally or attributable to other reasons. This raises questions about how socioeconomic factors may interact with other reasons for HAD, such as fear of discrimination. Considered through the lens of the HBM [42], or the BM [41], financial resources may constitute an additive *perceived barrier*, or a *predisposing factor*, for transgender people who may already be hesitant to engage with healthcare services due to fears of discrimination. However, it must be noted that most research is in the U.S. context, and financial associations with HAD may not be applicable to cohorts in countries with more financially accessible healthcare services.

Findings on associations with employment status, on the other hand, were mixed in the small number of related studies, with findings indicating both increased and decreased HAD for those who were employed. Additionally, significance of effects varied according to type of healthcare in one study, and gender in another. While findings indicating that unemployment is associated with higher HAD are consistent with other findings on financial factors, findings indicating that unemployment was associated with lower HAD are more difficult to parse. It could be that employment adds additional perceived barriers in accessing healthcare, such as difficulties in meeting healthcare appointments that fall inside of work hours. Findings in this area, however, draw from a small number of studies and further research is needed to disentangle these mixed findings.

The finding on differences in HAD between veterans and non-veterans indicates that veterans have lower levels of HAD due to non-suicidal self-injury. While this sole study investigated a very specific reason for HAD, this finding can also be considered alongside previous findings indicating an exception for associations between HAD and various factors in a sample of transgender veterans [9,46]. This speaks to a notable difference in patterns of HAD among transgender veterans, which may be explained, in part, by access to less financially burdensome Veteran's Administration healthcare.

Some studies found increased HAD to be associated with higher educational attainment, although conclusions are tentative due to data overlap. This finding runs counter to evidence from the general population, as higher levels of education typically predict greater healthcare utilization through financial channels [48]. It may be that financial benefits associated with higher educational attainment may not be as common in the transgender community, as unemployment is well documented to disproportionately affect the trans population [12], while employment discrimination and occupational minority stress are common barriers to employment for transgender graduates [12,49].

Findings on other identified demographic factors indicate limited association with HAD, often due to a lack of research using unique data sources and/or due to mixed evidence between studies. Such findings indicate gaps in our understanding of demographic variation in HAD and highlight the need for further research to bolster evidence in these areas. For example, findings suggest that relationship status may be associated with HAD due to financial factors, though significant effects came only from the NTDS dataset and were not replicated in the remaining study exploring this association. Additionally, there was some minor evidence for variance in HAD according to location, with findings from only two studies suggesting increased HAD in more urban environments. Conversely, there were no significant findings for association with other related factors of citizenship and demographic makeup of state, though again the body of evidence was very small, which prevents robust conclusions about these associations.

While evidence for associations indicate a direct link between HAD and such factors, additional interacting and confounding effects must not be overlooked. Other research has identified that, while sociodemographic factors may help to explain some differences in health-seeking behaviors in the trans community, some disparities persist beyond such characteristics. [11] As such, individual-level sociodemographic factors cannot fully explain disparities in HAD, and should be considered in conjunction with other factors such as external stressors and systemic barriers. Additionally, conceptual overlap between multiple factors, variation in findings for different reasons and settings of HAD, and potential confounding variables all speak to a greater need to understand how multiple factors interrelate in association with HAD. Firstly, understanding how possible overlap, such as the link between queer sexuality and younger age, or the link between older age and higher income, relate to HAD may further help to identify those most at risk of HAD. The importance of such interaction effects is further evidenced by variance in findings by other demographic variables such as gender. Future research should thus empirically explore interaction effects between demographic variables in relation to HAD.

Secondly, variation in findings, such as those related to gender identity, by operationalization of HAD indicates a gap in the research body in general. As associations with factors are very likely to be influenced by the reason for HAD, and by the type of healthcare being avoided, more research investigating HAD should consider measuring multiple reasons for, and settings of, HAD to allow for direct comparisons between such contextual factors. Additionally, a standardized measure of HAD integrating multiple contexts would be beneficial to research in this area.

Lastly, findings also identified potential confounding variables in associations that emphasize the need to explore additional contextual factors. Findings from one study indicate that several results were not maintained in multivariate analysis, though a very large number of covariates makes identification of potential confounders challenging. However, studies with smaller numbers of covariates give an indication of potential confounders requiring exploration in future research. For example, differences between bivariate and multivariate analyses in another study suggest a potential confounding effect of covariates of demographic variables, health access and status variables, and experiences of gender affirmation in healthcare settings, on the relationship between HAD and both gender and age. There was a similar finding for income in another study, where covariates consisted of other demographic factors, health status variables, and experiences of minority stress in healthcare settings. Still others confirmed confounding effects in analyses, such as a confounding effect of place of residence on the relationship between race/ethnicity and HAD, or a confounding effect of age and health insurance status, on the relationship between gender and HAD. Future research should further explore how such factors interact to constitute risk of increased HAD.

### 3.1 Limitations

Publication bias may be present in review findings, as only peer-reviewed studies were included. Additionally, only studies measuring demographic associations with HAD using inferential statistics were included. Studies reporting only descriptive statistics were omitted, and may provide further context into intra-community demographics related to HAD. Furthermore, heterogeneity and small number of studies precluded meta-analysis in most cases, while lack of access to further statistical data prevented meta-analysis in areas with sufficient number of studies and homogeneity.

## 4 Conclusions

Findings from synthesizing current research indicate that a wide range of demographic factors are associated with increased HAD, offering insight into subsections of the transgender community most at risk. Transmasculine gender identity and younger age had the most consistent evidence for association with increased HAD, while lower income and higher educational attainment also showed patterns of association with increased HAD. Findings for most other factors indicated mixed evidence, or associations investigated by only a small number of studies using unique datasets, highlighting areas requiring further investigation in future research.

## Funding

This research was supported by the 10.13039/501100002081Irish Research Council under award number GOIPG/2019/2511.

## Declaration of Competing Interest

The authors declare the following financial interests/personal relationships which may be considered as potential competing interests:

Siobhan D. Thomas reports financial support was provided by Irish Research Council.

## Footnotes

1 This calculation is an approximation, due to unreported final sample size following exclusion of participants in one study [31].
